# Screening mutations of *OTOF *gene in Chinese patients with auditory neuropathy, including a familial case of temperature-sensitive auditory neuropathy

**DOI:** 10.1186/1471-2350-11-79

**Published:** 2010-05-26

**Authors:** Da-Yong Wang, Yi-Chen Wang, Dominique Weil, Ya-Li Zhao, Shao-Qi Rao, Liang Zong, Yu-Bin Ji, Qiong Liu, Jian-Qiang Li, Huan-Ming Yang, Yan Shen, Cindy Benedict-Alderfer, Qing-Yin Zheng, Christine Petit, Qiu-Ju Wang

**Affiliations:** 1 Department of Otolaryngology/Head and Neck Surgery, Institute of Otolaryngology, Chinese PLA General Hospital, 28 Fuxing Road, Beijing 100853, China; 2 Beijing Institute of Genomics, Chinese Academy of Sciences, No.7 Beitucheng West Road, Chaoyang District, Beijing 100029, China Graduate University of the Chinese Academy of Sciences, 19A Yu Quan Rd, Beijing 100049, China; 3 Institut Pasteur, Inserm U587, Unité de Génétique et Physiologie de l'Audition, 25 rue du Docteur Roux, 75724 Paris Cedex 15, France; 4 Department of Biochemistry and Molecular Biology, Institute of Basic Medical Sciences, Chinese Academy of Medical Sciences and Peking Union Medical College, Beijing, China; 5 Department of Medical Statistics and Epidemiology, School of Public Health, Sun Yat-Sen University, Guangzhou 510080, China; 6 Department of Otolaryngology-HNS, Case Western Reserve University, Cleveland, Ohio 44106, USA; 7 Chinese National Human Genome Centre, Beijing 100176, China

## Abstract

**Background:**

Mutations in *OTOF *gene, encoding otoferlin, cause DFNB9 deafness and non-syndromic auditory neuropathy (AN). The aim of this study is to identify *OTOF *mutations in Chinese patients with non-syndromic auditory neuropathy.

**Methods:**

73 unrelated Chinese Han patients with AN, including one case of temperature sensitive non-syndromic auditory neuropathy (TS-NSRAN) and 92 ethnicity-matched controls with normal hearing were screened. Forty-five pairs of PCR primers were designed to amplify all of the exons and their flanking regions of the *OTOF *gene. The PCR products were sequenced and analyzed for mutation identification.

**Results:**

Five novel possibly pathogenic variants (c.1740delC, c.2975_2978delAG, c.1194T>A, c.1780G>A, c.4819C > T) were identified in the group of 73 AN patients, in which two novel mutant alleles (c.2975_2978delAG + c.4819C > T) were identified in one Chinese TS-NSRAN case. Besides, 10 non-pathogenic variants of the *OTOF *gene were found in AN patients and controls.

**Conclusions:**

Screening revealed that mutations in the *OTOF *gene account for AN in 4 of 73(5.5%) sporadic AN patients, which shows a lower genetic load of that gene in contrast to the previous studies based on other populations. Notably, we found two novel mutant alleles related to temperature sensitive non-syndromic auditory neuropathy. This mutation screening study further confirms that the *OTOF *gene contributes to ANs and to TS-NSRAN.

## Background

Auditory neuropathy (AN), also known as auditory dys-synchrony (AD), is a sensorineural hearing disorder and accounts for 7-10% of all childhood permanent hearing impairment[1]. The term was coined by Starr in 1996 to define a specific type of hearing deficit resulting from the impairment of auditory nerve function[2]. The primary lesion may be located in inner hair cells, in the auditory nerve, in the intervening synapse, or in any of the other neurons upstream of the auditory pathway. Clinical tests that lead to the diagnosis of auditory neuropathy include absence or severe abnormality of ABR, presence of the cochlear microphonic (CM) and/or OAE, absence of acoustic middle ear reflexes, and impaired speech perception much beyond what would be expected from the degree of hearing threshold elevation[3].

The clinical features of AN can vary considerably with respect to onset age, etiology, severity of hearing loss and the site of lesion. Auditory neuropathy has various causes, including neonatal insults (anoxia and hyperbilirubinemia), infectious processes (mumps and meningitis) and genetic factors[1,3-5]. However, 50% of patients with AN have no defined etiology[3,6].

AN was mapped to chromosome 2p22-p23 and the *OTOF *gene (MIM#603681) was subsequently found be responsible for that disease[7]. This 48-exon gene can encode short and long isoforms by means of alternative transcription start and splice sites. It has been linked with recessive non-syndromic deafness DFNB9[8] as well as non-syndromic recessive auditory neuropathy (NSRAN) [9]. The *OTOF *gene has been reported to be responsible for 2 to 3% of NSHL cases and seems to be more important to NSRAN cases in some populations[10,11], although there are other NSRAN-associated genes[12-15]. To date, more than 40 human pathogenic allelic variants of *OTOF *have been reported in familial or sporadic cases of AN worldwide and at least three quarters of these have been associated with NSRAN[11,16].

Mouse mutations of the otoferlin gene have yielded valuable insights into the molecular function of this gene[17,18]. Otoferlin, encoded by *OTOF*, is a FER-1-like protein and is critical for exocytosis at the auditory ribbon synapse. As the second member of a mammalian gene family related to *Caenorhabditis elegans gene fer-1*[19], this protein contains a transmembrane domain and six C2 domains, an overall structure supposed to perform a calcium-sensing function[20]. Otoferlin binds to the SNARE protein in a calcium-dependent manner, and SNARE is a highly conserved molecule vital for the release of neurotransmitters and for other events requiring fusion of membranes. During the neurotransmitter release at IHCs, otoferlin acts on mature vesicles and thus may regulate one of the final steps in the signaling pathway[21]. Therefore, identification of the underlying genetic variations in the *OTOF *gene is of particular importance for understanding the molecular pathway(s) for this complex neurological disorder.

Here we report *OTOF *gene re-sequencing from 73 AN patients in the Chinese Han population with a different mutation spectrum from other ethnic populations. In particular, we identified a patient affected with temperature-sensitive non-syndromic recessive auditory neuropathy (TS-NSRAN), whose phenotype was associated with a compound heterozygous expression of two mutant alleles in the *OTOF *gene.

## Methods

### Subjects and audiological evaluations

This study was approved by the Institutional Review Board of the Ethics Committee of China People's Liberation Army (PLA) General Hospital. Informed consent, blood samples and clinical evaluation were obtained from all human subjects. We enrolled 73 unrelated patients diagnosed as AN from January 1, 2003 to December 31, 2006, all of which are of Chinese Han ethnicity and residing in mainland China. The diagnostic criteria of AN[3] were defined as follows: (1) onset of auditory complaints prior to or during adolescence. Most of the patients had initial hearing loss at low frequencies and progressed to loss at all frequencies, accompanied by poor speech discrimination; (2) normal or partially normal transient evoked otoacoustic emissions (TEOAEs) and distortion product otoacoustic emissions (DPOAEs); normal tympanometry; and abnormal ABR thresholds and stapedial reflexes; (3) negative results on computerized tomography (CT) scanning of the temporal bone and magnetic resonance (MR) hydrography of the inner ear[15]. The case group was comprised of 32 females and 41 males, with onset age of hearing loss varying from infancy to adolescence ( = 14.6 ± 3.3) (Figure S1). Syndromic disorders were previously ruled out in all cases. The control group was consisted of 92 ethnicity-matched normal-hearing subjects.

### *OTOF *gene sequencing

Genomic DNA was isolated from whole blood of the subjects described above. Forty-five pairs of PCR primers for amplification of 48 exons and their flanking regions of the *OTOF *gene were designed using AgileBio Primer 3.0 online software and synthesized by Shenggong DNA Technologies (Table S1). PCR reactions were carried out in a total volume of 25 μl containing 5 × 10^-6 ^mmol/l (5 μM) of each deoxynucleotide triphosphate (dNTP), 1 × 10^-8 ^mmol/l (10 nmol) of each primer, 2.5 U of AmpliTaq Gold polymerase, 2.5 μl of 10 × TE buffer and 100 ng of genomic DNA. PCR amplifications were carried out in PE9700 thermocyclers (Applied Biosystems), using conditions of 5 min at 94°C, followed by 30 cycles of 30 sec denaturation at 94°C, 30 sec annealing at 60°C, 30 sec extension at 72°C and then 7 min extension at 72°C. Conditions were modified slightly to improve the amplification of some exons. Each PCR product (2 μl) was first separated on a 1.5% agarose gel, then purified using Millipore filter plates and finally sequenced by ABI 3730Xl DNA Analyzer, with using the same primers above, which result was then analyzed by DNAStar software (DNASTAR, Madison, WI). Mutations were detected by aligning the sample sequences with reference sequences (NM_194248.1) from the GenBank database.

## Results

### Mutation screening identified 5 possibly pathogenic and 10 non-pathogenic sequence variants

**Figure 1:**
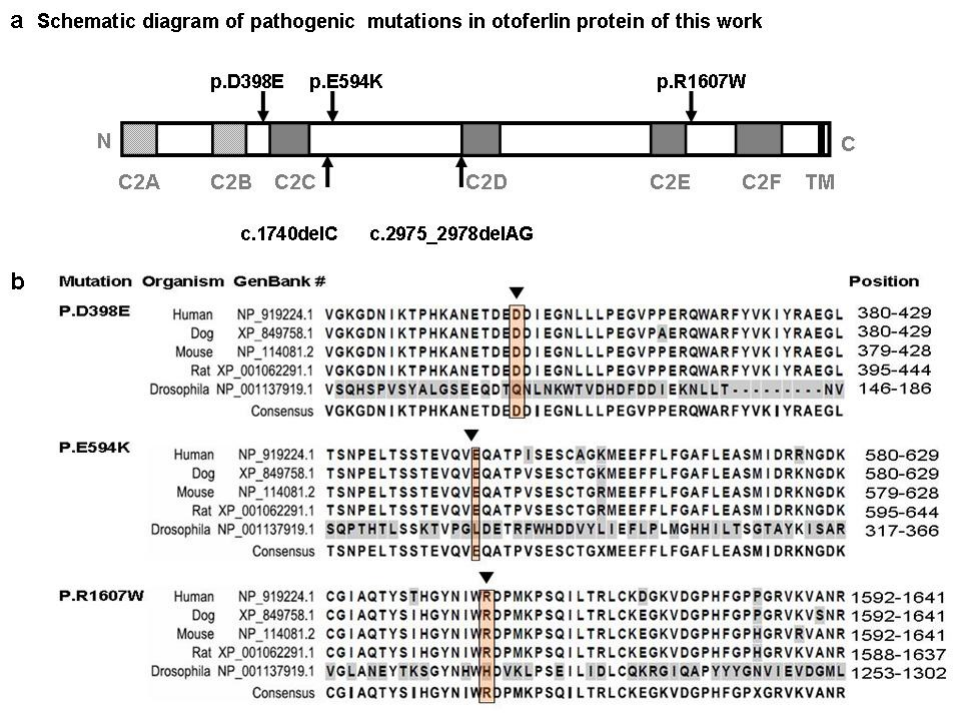
**Novel pathogenic *OTOF *mutations in this work**. **a**. A schematic diagram of the human otoferlin structure with all pathogenic mutations of this work illustrated (shown by arrows)[9,20]. **b**. ClustalW alignment result of the long isoform of otoferlin among different organisms, including human, dog, mouse, rat and drosophila*. The results as shown are the residues including 3 missense mutations reported in this work with their flanking regions. Different residues are shown in gray. Locations of mutated amino acids are framed in orange boxes and pointed by arrows. * Different from mammals having multiple ferlin genes, drosophila only has one ferlin gene called misfire, which is also its protein name[28]

**Figure 2:**
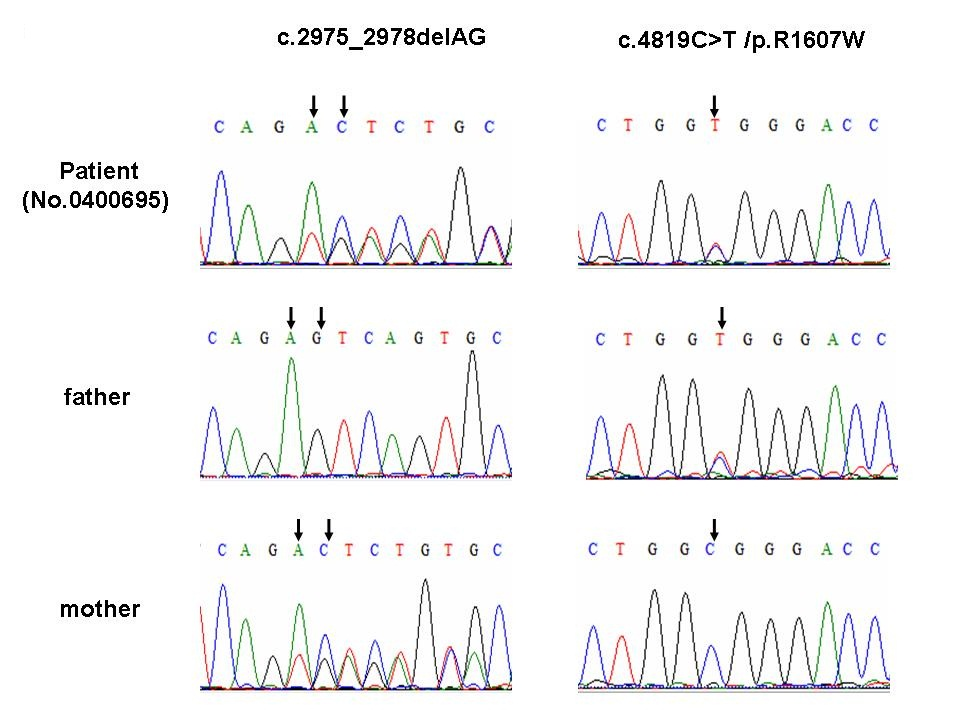
***OTOF *sequence analysis of a TS-NSRAN family at mutation sites**. We found 2 heterozygous mutations in the *OTOF *gene of the patient No.0400695, which are c.2975_2978delAG (p.Q994VfsX6) and c.4819C > T (p.R1607W), inherited from the mother and father respectively. Corresponding mutation locations are indicated by arrows

**Figure 3:**
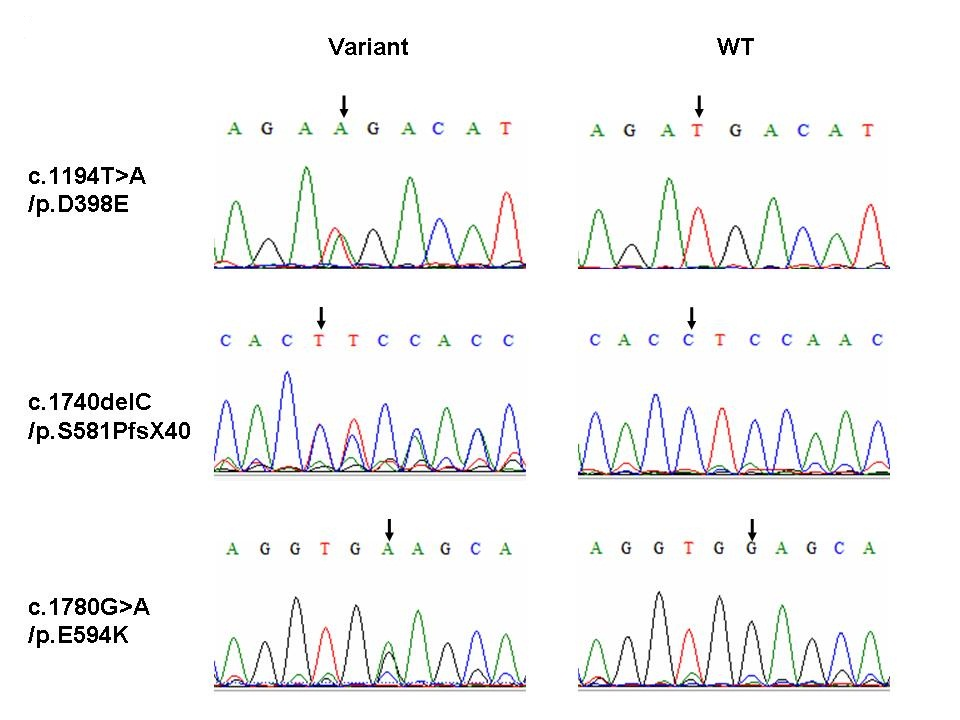
**Sequencing chromatograms of pathogenic variants identified in this work**. Sequencing chromatograms of pathogenic variants c.1194T > A/p.D398E, c.1740delC/p.s581PfsX40 and c.1780G>A/p.E594K. Two columns are sequencing chromatograms of variants carriers and control. Corresponding variants locations are indicated by arrows

Upon screening the *OTOF *gene sequence of 73 AN patients, five novel variants were found in four patients of the case group that could be possibly pathogenic (Table 1, Figure 1), including 2 frameshift mutations and 3 missense mutations: (a) c.1740delC (p.S581PfsX40), a cytosine deletion at position 1740 (exon 16) which introduced a stop codon and caused premature termination of otoferlin at position 621; (b) c.2975_2978delAG (p.Q994VfsX6), a deletion of AG at exon 25 which led to a protein sequence change from position 994 and consequently a premature termination codon at position 1000; (c) c.1194T > A (p.D398E), the consequence of a transversion from T to A at DNA sequence position 1194, which caused a substitution from aspartate to glutamate in exon 13; (d) c.1780G > A (p.E594K), a transition from G to A at nucleotide 1780 which resulted in a substitution from glutamate to lysine in exon 16; and (e) c.4819C > T (p.R1607 W), a transition from C to T, which resulted in the amino acid arginine (in codon 7 of exon 40) being replaced by a tryptophan. (Figure 2) Apart from c.2975_2978delAG (p.Q994VfsX6) and c.4819C > T (p.R1607 W), which were identified in one patient as compound heterozygous mutations, (Figure 3) the other three were heterozygously carried by three patients, respectively. We assumed these varients were possibly pathogenic because (1) both frameshift mutations yielded a premature stop termination codon and the 3 missense mutations occurred in regions of the otoferlin sequence that are highly conserved among mammalian species (Figure 1) and (2) none of these mutations were observed in the set of 92 unrelated controls (184 chromosomes).

**Table 1:** Pathogenic sequence variants identified in this study

Exon	DNA level	Protein level	Consequence	Subject	Age of Onset (year)	Control chromo-some
13	c.1194 T > A	p.D398 E	Missense	0501207	19	0/184
16	c.1740delC	p.S581PfsX40	Frameshift	0501800	0	0/184
16	c.1780 G > A	p.E594K	Missense	0501768	12	0/184
25	c.2975_2978	p.Q994VfsX6	Frameshift	0400695	1	0/184
	delAG					
40	c.4819C > T	p.R1607W	Missense	0400695	1	0/184

*Nomenclature of mutations is based on GenBank reference sequence accession number NM_194248.1. The nucleotide 1 is its first nucleotide of the translation initiation codon.

Moreover, 10 variants encoding polymorphic changes were identified, including 4 missense mutations: c.145C > T (p.R49W), c.157G > A (p.A53T), c.158C > T (p.A53V) and c.244C > T (p.R82C). Some of these polymorphisms have been previously reported in other populations (Table S1) [11,16,22,23].

### TS-NSRAN case: its clinical features and *OTOF *gene mutations

This exceptional case was a 7-year-old boy (Subject No. 0400695) showing onset of hearing disorder at 13 months after birth. Three years of following this patient indicated that the boy had periodic hearing loss, which was particularly salient when he suffered fever, at which times he would lose communication ability and was characterized by normal otoacoustic emission, but no evoked ABR. The observation that the boy's audiological evaluation correlated with his body temperature (over a 15-day period in our hospital when he was four years old) led to his diagnosis of temperature-sensitive AN. When his body temperature was below 36.5°C, the audiological tests showed a flat audiogram (40-60dB HL), normal DPOAE, type A tympanogram, negative results of temporal bone CT and MR hydrography of the inner ear, and absence of ABR and acoustical stapedial reflex. However, his hearing was affected by a slight change of body temperature. His mother found that his hearing in the morning is generally better than in the afternoon, and temperature measurements showed that his body temperature in the afternoon was generally 0.1-0.6 Π higher than that in the morning. Correspondingly, his pure tone thresholds increased 10dB HL on average speech frequencies. When his body temperature rose above 36.5°C, the boy's hearing loss was severe (70-80dB HL) and this symptom could last for a whole day.(Table 2) For clinical details of this patient, see our previous report [24].

**Table 2:** Audiological data summary of NSRAN case

Pure tone test (Hz dB HL)										
Body temperature		250	500	1000	2000	4000	8000	Average auditory threshold	ABR	DPOAE
36.0°C	L	45	45	40	50	70	75	51		
	R	40	45	65	65	55	65	58		
36.5°C	L	55	60	65	85	85	105	74	No Response	Normal
	R	60	55	65	70	70	65	65		
36.9°C	L	60	80	75	80	90	100	81		
	R	65	70	75	75	85	70	76		

Sequencing results revealed two mutations of the *OTOF *gene in the patient, namely c.2975_2978delAG (p.Q994VfsX6), and c.4819C > T (p.R1607W). The frameshift mutation (c.2975_2978delAG) was found in the patient's mother and the missense mutations (c.4819C > T) came from the patient's father. Both parents, heterozygote mutation carriers (Figure 2), have normal hearing. The two mutant alleles of c.2975_2978delAG (p.Q994VfsX6) and c.4819C > T (p.R1607W) were not found in our control group.

## Discussion

Han Chinese population takes about 20% of the entire global population, and distributes mainly in China. This population has its unique genetic background, and that is why Han Chinese in Beijing (CHB) was chosen by the international HapMap project as one of the four populations for DNA analysis, the result of which http://www.hapmap.org/ also showed the great variation between CHB and the other three populations. So far, the screen of *OTOF *gene has been preformed on Spanish, Caucasians, Pakistani and Brazilian populations[10], however, it is not well studied on Han Chinese population.

In the present study, we performed a mutation screening of the *OTOF *gene within 73 unrelated AN patients and 92 controls in Chinese population. Of those, 5 novel possibly pathogenic variants were identified in four AN patients with the mutation frequency of 5.5% (4 of 73) and revealed a quiet different mutation spectrum and a comparatively lower genetic load of that gene in Chinese AN patients. Surprisingly, none of the pathologic allelic variants reported previously were found in the collected Chinese Han sample, including the most frequently reported one, Q829X, which was shown to be responsible for at least 3% of NSHL cases in some populations[11,22,23,25,26]. Close scrutiny of the previous reports revealed that most of the studies were based on Spanish, Caucasian and Pakistani populations with the mutation frequencies varying from 5.1% to 3.1% to 2.3% respectively[11,23], which proportion was even higher in NSRAN cases[10,11]. According to haplotype data of the international HapMap project, the CHB displayed a notable difference from the other three groups at the 2p22-p23 loci where the *OTOF *gene is located. These data suggested that great discrepancies in the mutation spectra of the *OTOF *gene could be observed in different ethnic populations. Our perception is supported by two recent study that reported in Pakistani[16] and Brazilian[10] populations, in which was found a quite different mutation spectrum from those previously reported as well.

Another striking finding in our study was the occurrence of two novel mutant alleles in the TS-NSRAN patient, thus being the second reported case of the temperature-sensitive phenotype related to *OTOF *gene mutations in the literature. Temperature-sensitive auditory neuropathy (TS-AN) was first reported in 1998[27], and the responsible heterozygous mutation of the *OTOF *gene, p.I515T, was first found in two affected children and their father[23], but no available information on their maternal mutation was in the study. However, in our study, the patient's two mutant alleles were clearly identified from both parents. The mutation of c.2975_2978delAG led to a premature stop codon that was transmitted from the maternally inherited mutation, and which could trigger the nonsense-mediated decay (NMD) response. Even if the truncated protein could be produced, it could not function well, because the c.2975_2978delAG occurred at the beginning of the C2D domain and the last four C2 domains are predicted to be critical for protein function[8]. The paternally inherited mutation p.R1607W is a missense mutation, and p.R1607W was the consequence of replacement of an alkaline arginine to a neutral tryptophan. The manifestation of TS-AN described here may be caused by a cumulative effect of the frameshift combined with the missense mutations. The combined effect of the two mutations could interfere with normal production of otoferlin protein.

Excepting NSRAN patient, each of the other three patients with pathogenic variants (Table 1) carries only one copy of an *OTOF *variants, which we assume are causative factor for their disease. In previous study, there is familial case linked to OTOF gene but with single heterozygous variant[9]. Moreover, among the recessive hereditary hearing loss genes, the high prevalence genes of GJB2 or SLC26A4 also can be found only one mutant allele in the congenital hearing loss or enlarged vestibular aqueduct syndrome patients. Multi-gene related disease and epigenetic imprinting could be two possible explanations. Further studies to elucidate the compound heterozygous mutation interactions and the role of those possibly pathogenic variants to *OTOF *gene and AN are worth conducting. Additionally, considering the comparative lower genetic load of the *OTOF *gene, other genetic or environmental etiology of AN should be searched in Chinese population.

## Conclusions

In conclusion, in 73 Chinese patients with diagnosed AN and in a control group of 92 unrelated people with normal hearing, a mutation screening of the *OTOF *gene identified 5 possibly pathogenic variants associated with NSRAN and 10 non-pathogenic variants. Further investigation of a boy affected with TS-NSRAN revealed two mutant alleles: c.2975_2978delAG (p.Q994VfsX6) and c.4819C > T (p.R1607W). The special mutation spectra of the *OTOF *gene, revealed in the present study and differing from previous ones, could be the result of across-race diversity of this gene.

## List of Abbreviations

ABRs: auditory brainstem responses; AN: auditory neuropathy; AR: abnormal acoustic reflex; CMs: cochlear microphonics; DPOAEs: distortion product otoacoustic emissions; IHC: inner hair cell; NMD: nonsense-mediated mRNA decay; NSRAN: non-syndromic recessive auditory neuropathy; OAEs: otoacoustic emissions; TEOAEs: transient evoked otoacoustic emissions; TS-AN: temperature-sensitive non-syndromic auditory neuropathy; TS-NSRAN: temperature-sensitive non-syndromic recessive auditory neuropathy.

## Competing interests

The authors declare that they have no competing interests.

## Authors' contributions

The work presented here was carried out in collaboration between all authors. QJW defined the research theme, designed methods and experiments. DYW carried out the laboratory experiments, analyzed the data. DYW and YCW interpreted the results and prepared the paper. DW, YLZ, JQL and YBJ co-designed the dispersal and colonization experiments. SQR, LZ and QL co-worked on associated data collection and their interpretation. QJW, CP and QJW discussed analyses, interpretation, and presentation. QYZ, HMY and YS conceited and coordinated the study. All authors have contributed to, seen and approved the manuscript.

## Pre-publication history

The pre-publication history for this paper can be accessed here:

http://www.biomedcentral.com/1471-2350/11/79/prepub

## Supplementary Material

Additional file 1 **Supplemental Tables**. PCR primers for *OTOF *gene screening and non-pathogenic sequence variants identified in this study. Click here for file

Additional file 2 **Supplemental Figures**. Age-of-onset distribution of the entire case group and audiometric test results of NSRAN case. Click here for file
