# Supplementary material for: Screening mutations of OTOF gene in Chinese patients with auditory neuropathy, including a familial case of temperature-sensitive auditory neuropathy
Source: BMC Med Genet. 2010 May 26;11:79. doi: 10.1186/1471-2350-11-79 (PMC2901213; doi:10.1186/1471-2350-11-79)
Supplement: Additional file 1 — Supplemental Tables. PCR primers for OTOF gene screening and non-pathogenic sequence variants identified in this study. [file 1471-2350-11-79-S1.DOC]

**Figure S1 Age-of-onset distribution of the entire case group**


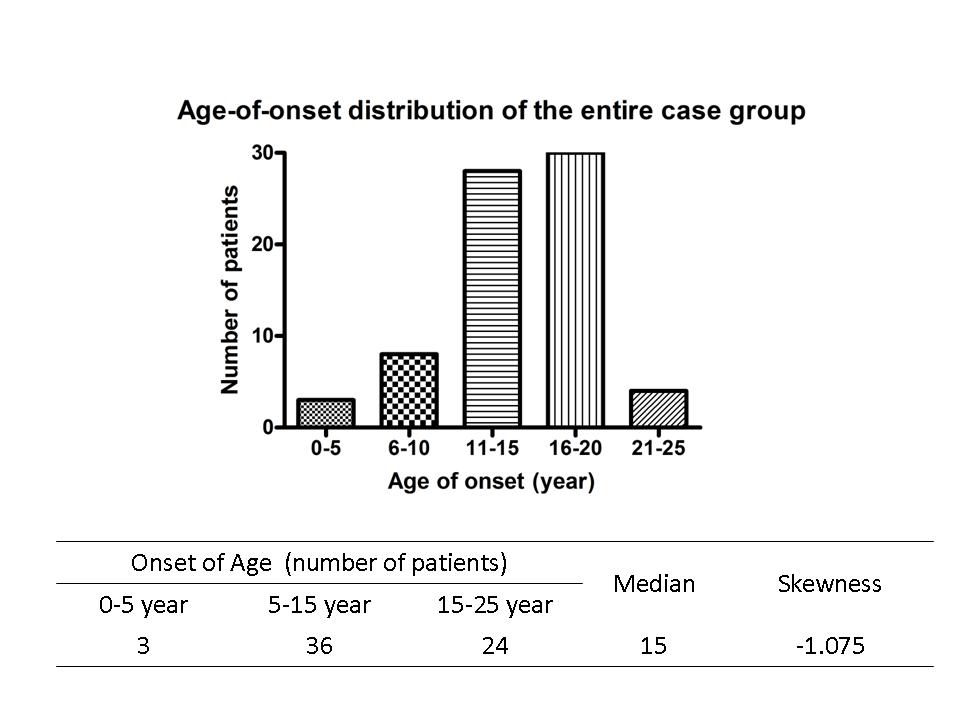


**Figure S2 Audiometric test results of NSRAN case**


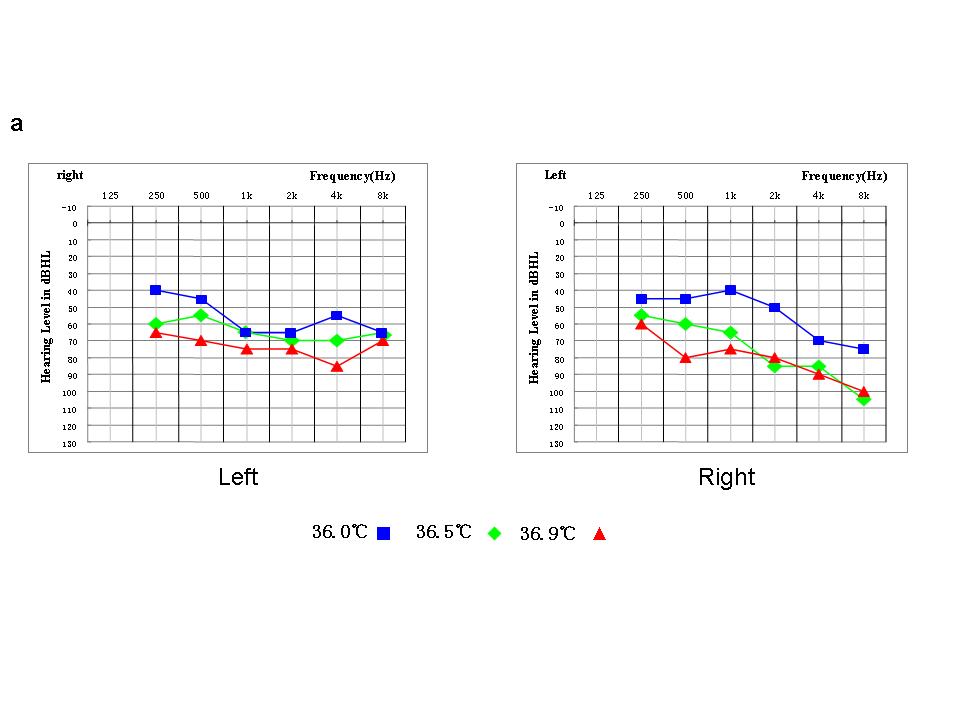


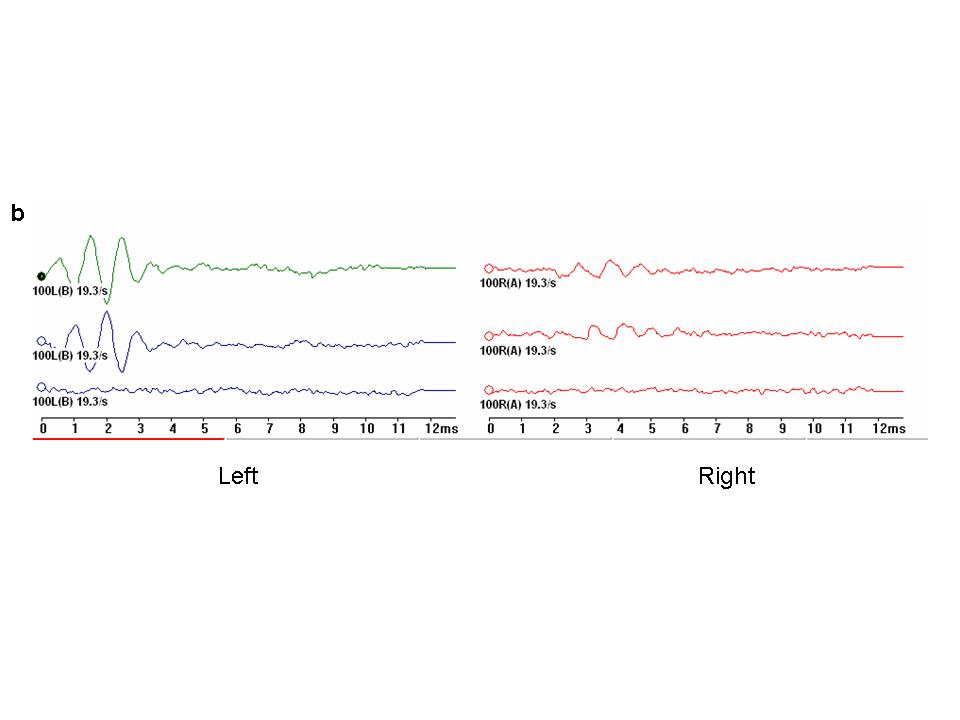


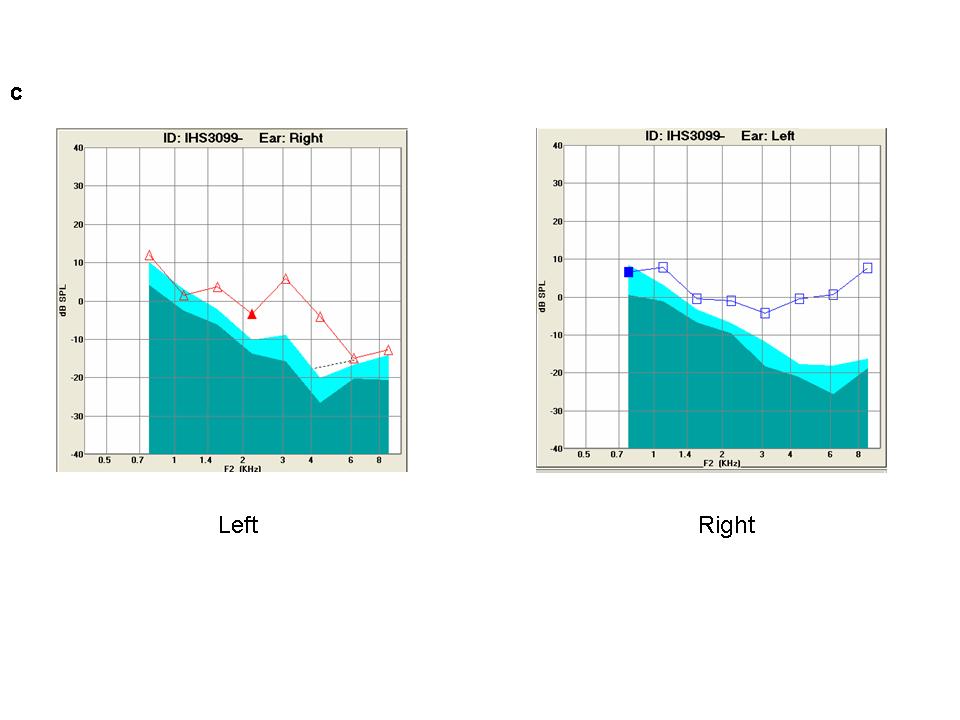


**a.** Pure tone test results of NSRAN case under different body temperature. Axillaty temperature was measured at the mean time of pure tone test producing. The blue squares, green diamonds and red triangles are respectively representing average auditory threshold at 36.0 ℃, 36.5 ℃ and 36.9 ℃. **b.** ABR results of NSRAN case.

The bilateral test showed negative result. **c.** DPOAE result of NSRAN case.

The result shows bilaterally normal DPOAE response at each frequency.
