# Supplementary material for: Screening mutations of OTOF gene in Chinese patients with auditory neuropathy, including a familial case of temperature-sensitive auditory neuropathy
Source: BMC Med Genet. 2010 May 26;11:79. doi: 10.1186/1471-2350-11-79 (PMC2901213; doi:10.1186/1471-2350-11-79)
Supplement: Additional file 2 — Supplemental Figures. Age-of-onset distribution of the entire case group and audiometric test results of NSRAN case. [file 1471-2350-11-79-S2.DOC]

## Table S1 PCR primers for OTOF gene screening

|  | | | | |
| --- | --- | --- | --- | --- |
| **Primer set** | **Exon amplified** | **Forward primer (5’-3’)** | **Reverse primer (5’-3’)** | **Product size (bp)** |
| 1 | 1 | AGAGAAGAGAGAGGCGTGTGAG | GGCAGATGACTACCTGTGAAAAG | 323 |
| 2 | 2 | CACGAGGTCCCATGTTGCA | CGGCCAGTGCCTGGGATT | 295 |
| 3 | 3 | AGGTGGTTGTTGGAACTAAATGA | GAGTGTAGGTCCCCTTTTTAAGC | 372 |
| 4 | 4 | CCAAGCAGTCACAGCCCTT | ACCTCGCCATGCTTGAGAG | 302 |
| 5 | 5 | TCCAGTGAGGCAAGGGTGT | CTTGGATGTCTCTCCAGAAG | 434 |
| 6 | 6 | TCTGCAGACCTAGGCTTGC | CGACAGCCCACTCCTGAG | 333 |
| 7 | 7 | ATCTGTCTCGGGACCAGTAGGT | TAGGGCGTCTCCTTCCTAGAG | 377 |
| 8 | 8 | TAACTCTCAGCTTTCTGGATG | TACCCAAATTCCAATCATGGC | 303 |
| 9 | 9 | CTCTCTGTTACTTCTGCCTT | TTCTTTGGGTTTGTATCTCT | 461 |
| 10 | 10 | GACACTTGAGGCATAGAAGGAGA | AAGATGTGGCTCTGTTTGTCAGT | 500 |
| 11 | 11 | GGTCAATCAGGATCAGATGAGTT | GCTGCCTCTTTATCATGGGTCTA | 335 |
| 12 | 12 | GTAAACAGTGGCCAGCAAGAA | TGTGTGTACTAACAGTCGCCAGA | 399 |
| 13 | 13 | ACTCTAGGGACCAAGACAGCATT | GACTGATATTCTCAGCCATCCTC | 467 |
| 14 | 14 | GTGCCAGGACCCAGGAGT | TGATTTCCAGCCTTGTCTTAC | 387 |
| 15 | 15 | ATGCCACGCCCTCACCT | CAAATCCTCACCCTCCC | 394 |
| 16 | 16 | CTGAATGGCACACATGAAGTTCT | GCCTTATCCTGAGGTATGACTCC | 484 |
| 17 | 17 | CGGCCTGTCTGTGAGACG | GAGCCTCACACTTACCACC | 290 |
| 18 | 18&19 | CTAATGATGTGTCACGTCTGAGC | GAGAATGGGAACAGAGGTCCTG | 669 |
| 19 | 20 | ATTCTGGAGTGACGTCAGGATCT | CAGGGGCTCTGTAGATTCTTCCT | 575 |
| 20 | 21 | TGATCAAAACGGAGAAGTCCTAC | AGCTTCTGCAGGAAGTTCTGG | 531 |
| 21 | 22 | GAGGGCCTGGTTGTGAGAAG | GAGCCCCCTGATCCTGAG | 284 |
| 22 | 23 | CCTCCTTCGCAGGAAAACAT | CCTGTCACTCAGGCTTCCAG | 497 |
| 23 | 24 | AGCGGGATGGCTGTGAAG | CAGGTGAGGCTTCGAGTGAG | 346 |
| 24 | 25 | GCGGGATGGCTGTGAAG | CTGTCGGCGGCAAAGAG | 464 |
| 25 | 26 | TGCCTTCCCACCCGTCAG | AGCCCGTAGCCTTTCCAG | 440 |
| 26 | 27 | CACACCTGTCCCTTACAACAACT | CTTTGGTCAGGATGGGTAGGAG | 551 |
| 27 | 28 | CAGATCATGGCAAACAACTCAT | GGGATGACAAGCCACTTCC | 279 |
| 28 | 29&30 | GTTCCCTATTGGATGGTCCAG | CACCAGGGTGACCTTCTTCTAT | 666 |
| 29 | 31 | CTTTAGTTCTCCGCACCTTCCT | TAGTCTGGTTCACAGGATGTGTC | 296 |
| 30 | 32 | AAAGAAACATCCTCACTCAGACC | CAGCTCTTTTCCACTGCATCTTA | 388 |
| 31 | 33 | GGCACCTTCTCCATAAAATAAGG | GAGTTGGGATCCAGCTCTTG | 394 |
| 32 | 34 | TCTAGTGCATTAAGACTGGTCTGG | ATGCTGCTTCCCTGGCTACTAA | 298 |
| 33 | 35 | GTAAAGCAAATTAGCGACAGAGC | CTTTGGGGTATACCTGAGACAGA | 372 |
| 34 | 36 | AGCCTAGGAGCAGACCCTTCTA | TTAACTGCTGAGTCATGGGAGAG | 397 |
| 35 | 37 | CTACACCCTGACCCACATTTGTCTT | CCAGATGGGAGGATGAGGAGACTT | 222 |
| 36 | 38 | CTTCACTGTCATAGGACCCTCAG | TCTCAAATCACCAGGATCTGAAT | 587 |
| 37 | 39 | CTCCCACCCTAGCCAATCCTTAA | TGGAGGCAAAGCAGGCACACT | 273 |
| 38 | 40 | GCAACAGCCAACTTTAGAGAACT | CAGAGGGTCACTAAGACCAGGTT | 396 |
| 39 | 41 | CTAAGTCCCTAGTCCCAGCAAAG | CTTCTACCTTTACTGACTCAGGT | 298 |
| 40 | 42 | TCCTTGACAGAACAAAGGTTAGG | TATCGATTGGTTGGTCAGTTTCT | 361 |
| 41 | 43 | CAACCCCAAACCACTCCTC | CTGATGGACTGGAAGCAATGAC | 764 |
| 42 | 44 | GGCTGCGGGTCTGGAGATGT | CCCATCCCTGCCCTGCTCT | 296 |
| 43 | 45&46 | GTCTGCACTCAGCAAACAAGG | ATCGTCTCCTTCCTGTTCCAG | 556 |
| 44 | 47 | CAGAGGGGTGCCTAGAACTG | GTGTGCTTGAGTGCATGTGT | 500 |
| 45 | 48 | TGACCGGCTCCTCTCTTCCACTT | GGGAAAGAGTCCAAGCCACTGAAA | 279 |
| Because of the short distance, exons 18 to19, 29 to 30, and 45 to 46 were amplified as three large regions. | | | | |

## Table S2 Non-pathogenic sequence variants identified in this study

| **Exon** | **DNA level** | **Protein Level*** | **Genotype**  **(-zygous)** | **Cases**  **(Chromosome)** | **Frequency**  **(%)**** | **Controls**  **(Chromosome)** | **Frequency**  **(%)***** | **References** | **dbSNP rs#** |
| --- | --- | --- | --- | --- | --- | --- | --- | --- | --- |
| 1 | c.76C>A | p.R26R | Hetero | 1 (1) | 0.7 | 0 (0) | 0 | this work |  |
| 2 | c.129C>T | p.D43D | Hetero | 16 (16) | 10.9 | 18 (18) | 9.8 | this work |  |
| 3 | c.145C>T | **p.R49W** | Hetero | 2 (2) | 1.4 | 4 (4) | 2.2 | this work |  |
|  | c.157G>A | **p.A53T** | Hetero | 1 (1) | 0.7 | 1 (1) | 0.5 | this work |  |
|  | c.158C>T | **p.A53V** | Hetero | 18 (18) | 12.3 | 26 (26) | 14.1 | [16, 23] | rs1879761 |
| 4 | c.244C>T | **p.R82C** | Hetero | 13 (13) | 8.9 | 13 (13) | 7.1 | [11, 16, 22, 23] | rs13031859 |
| 5 | c.372A>G | p.T124T | Homo  /Hetero | 25/34 (84) | 57.5 | 30/39 (99) | 53.8 | [11, 16, 22, 23] | rs11687696 |
| 39 | c.4677G>A | p.V1559V | Hetero  /Heteros | 1/13 (15) | 10.3 | 2/18 (22) | 11.9 | [16, 23] | rs2272071 |
|  | c.4767C>T | p.R1589R | Hetero | 11 (11) | 7.5 | 15 (15) | 12.3 | this work |  |
| 44 | c.5418C>T | p.L1706L | Hetero | 5 (5) | 3.4 | 8 (8) | 4.3 | this work |  |

*Missense variants are shown in bold.

**Incidence in 73 case-group individuals, number of chromosome (146 in total).
